# Attenuating the Negative Impact of Unemployment: The Interactive Effects of Perceived Emotional Intelligence and Well-Being on Suicide Risk

**DOI:** 10.1371/journal.pone.0163656

**Published:** 2016-09-29

**Authors:** Natalio Extremera, Lourdes Rey

**Affiliations:** 1 Department of Social Psychology, University of Malaga, Malaga, Spain; 2 Department of Personality, Evaluation and Psychological Treatment, University of Malaga, Malaga, Spain; Wuhan University, CHINA

## Abstract

A growing body of research has demonstrated that deficits in well-being may be related to increased suicide risk, but there is only a limited number of studies that have focused on specific protective factors that can serve as a buffer against suicidal ideation and behaviours. Given that unemployment may be a factor leading to increased risk for suicide, this study assessed whether perceived EI might be a potential moderator in the relationship between life satisfaction/happiness and suicidal behaviours in a relatively large sample of unemployed individuals. Participants were 1125 unemployed (506 men and 619 women) who completed satisfaction with life and happiness questionnaires, the Suicidal Behaviours Questionnaire and the Wong and Law Emotional Intelligence Test. Consistent with the interaction hypothesis, lower scores in life satisfaction and happiness were associated with higher levels of current suicidal behaviours, and perceived EI scores moderated these relationships. Interventions targeting well-being via the promotion of emotional abilities may be useful in the prevention of suicidal ideation in the unemployed. The implications for these findings for research and practice are discussed.

## Introduction

While all European countries have been affected by the economic crisis, the adverse consequences of recession in Spain have been among the worst in term of job losses and unemployment [1]. In particular, in 2016, Spain has the second highest unemployment rate in the European Union after Greece [2]. Accordingly, recent studies have highlighted that economic crisis has significantly increased the frequency of mental health disorders, particularly among Spanish families experiencing unemployment [3,4]. Indeed, a bulk of meta-analytic research has previously documented that unemployment represents a period with many stress-related consequences and a high risk to well-being and quality of life [5,6]. For example, unemployment has also been associated with deleterious health problems, such as depression or psychopathologies [7], low levels of self-esteem [8], a significant increase in physical complaints, fatal injury and mortality [9,10] and with strongly negative effects on life satisfaction and happiness [11,12]. Changes in social status, time-structure, disruptions in both work and family roles, loss of self-concept and identity, decreasing social contacts, subsequent financial deprivation, and uncertainty about the future have been attributed as possible sources of pervasive adverse consequences for people’s experience of well-being [13,14]. Accordingly, most studies of psychological well-being have revealed that the unemployed are relatively unhappy and show high levels of mental distress compared with their employed counterpart [11,15]. For example, unemployed report lower levels of life satisfaction than their employed peers [5,16]. Thus, longitudinal studies have shown that although life satisfaction is moderately stable over time, individuals reacted strongly to unemployment and did not completely return to their former levels of satisfaction, even after they became reemployed [11]. In sum, these findings suggest that unemployment has a strong influence on long-term levels of individuals’ well-being [5].

On the other hand, the available literature documents numerous emotional and behavioural consequences for dissatisfied people, including depressive disorder [17], anxiety [18], and disease mortality [9]. Similarly, a number of studies have confirmed that self-reported deficits in well-being are important predictors of long term health hazards, such as suicidal behaviours, ideation and attempts. For instance, prior work has revealed that indicators of well-being, such as life dissatisfaction and low subjective happiness, have been shown to predict overall and injury-related mortality in healthy adults [9,19], as well as suicide and fatal injury in adults unselected for health status in a 20-year follow-up [10,20] suicidal attempts among in-patients with schizophrenia [21]. More interestingly, research on the link between labour market status, well-being indicators and suicide have found a significant association between happiness and suicide indicators, particularly for unemployed people [15]. However, low happiness or high levels of life dissatisfaction do not account for all the individual variability in experienced suicidal thoughts and behaviours. The presence of personal resources -emotional skills that indicate positive psychological functioning, such as emotional intelligence-, might explain to some extent the variability in the unhappiness–suicide outcomes [22].

Emotional intelligence (EI) might be one factor to serve a role in the relationship between low well-being–suicide outcomes. EI is a psychological construct that has attracted particular attention in the psychosocial literature due to its positive outcomes in the realm of subjective well-being, interpersonal functioning and health behaviours [23]. From an ability approach, EI is defined as a set of skills, such as the ability to perceive emotions, to access and generate emotions so as to assist thought, to understand emotions and emotional knowledge, and to reflectively regulate emotions so as to promote emotional and intellectual growth [24]. Much of this research has focused on EI as a predictor of health related correlates [25,26,27], focusing on the promising role of EI in explaining how people cope with and adapt to stressful situations contributing to psychological well-being and health [28]. As a factor that helps with handling negative emotions and reducing stress, the skills that comprise EI might play a key role in curbing suicidal ideation and attempts [29]. As researchers have suggested, the high EI individual, relative to others, is more apt to engage in problem-solving behaviours, and avoids self-destructive, negative behaviours such as smoking, excessive drinking, drug abuse, or violent episodes with others [30]. Accordingly, Cha and Nock hypothesised that these emotional skills might play a vital role in helping to understand and alleviate suicidality, finding preliminary support for the significant role of EI as a protective factor to decrease the likelihood of suicidal ideation and attempts among those adolescents at risk [31]. Similarly, other researchers found that EI abilities incrementally explained suicidal ideation in a sample of college students, even after controlling for demographic variables, personality traits, affectivity, and cognitive intelligence [32]. In summary, it is plausible to think that unemployed individuals experience intensive and negative affective states which might contribute to suicide risk. Ability EI model conceptualized it as a set of interrelated abilities to process information of an emotional nature, that is, EI is conceived as abilities to engage in efficiently information processing about one’s own and others’ affective states and the ability to use this information as a guide to thinking and behavior [24]. From this approach, unemployed with higher EI are also better at perceiving and understanding their own psychological state, which can include managing negative moods effectively and being less likely to suffer from suicidal ideation. In other words, unemployed having emotion skills articulated in the EI theory should be more adaptable to withstand and regulate these negative affect and the emotional skills an individual uses in response to life dissatisfaction are more likely to increase or reduce their vulnerability toward suicidality [33]. Nevertheless, there are still considerably fewer published studies on the moderator role of EI in the link between deficits in well-being and suicide. One valuable exception is the work of Ciarrochi et al., which revealed that EI abilities showed a moderating effect on the relationship between stress and mental health outcomes, including suicidal ideation in university students [34]. A typical limitation of previous studies on EI and suicidal behaviours is that most studies on this topic relied predominantly on college and adolescent students. In our study, the unemployed sample was chosen because, for most people, unemployment is perceived as a particularly stressful situation that has been found to be a suicide risk factor [35]. In view of the wide range of stressful situations reported by this experience and given that unemployment may be a factor leading to increased risk for suicide, it would seem important to understand the role EI abilities in the well-being outcomes–suicide link in this sample. Since different meta-analyses have shown that EI abilities promote healthy perceptions, mental health, and psychological and physical well-being [36,37,38], understanding the role of potentially modifiable personal dimensions, namely EI, in the link between reduced well-being and suicide risk, might be useful in designing psychosocial interventions with the aim of decreasing the risk of suicidal behaviours in people who are less satisfied with regard to different life domains. Despite that the above-mentioned studies have shown that suicide risk is related to and can be predicted by deficits in well-being indicators and EI, no studies have specifically examined the joint contribution of well-being indicators and EI abilities to suicidal ideation during unemployment. Some researchers have underlined the importance of integrative psychosocial models and a need for more scientific work examining the importance of personal resources that are closely associated with psychological health in unemployment and the mediating or moderating relationships between psycho-social resources, coping, and well-being outcomes [5]. Investigating the specific joint contribution of deficits in emotional abilities and well-being that relate to suicidal ideation might help the design of more effective program interventions that mitigate negative psychological effects of job loss. Besides, these empirically derived and integrative psychosocial models might be meaningfully integrated in the development of employment promotion programs to enhance well-being and facilitate a successful return to the work.

The present study was designed to broaden our understanding of this relationship by examining the potential moderator effects of EI in a relatively large sample of unemployed individuals. To date, few studies have examined the links between reduced well-being and suicidal behaviours and no study has examined the interactive effect of reduced well-being and EI on suicidal behaviours. Given the aforementioned considerations, the purpose of the present study was twofold. The first purpose was to examine relationships among life satisfaction, subjective happiness, EI and suicide risk in a sample of unemployed individuals. Second, as mentioned above, there are both theoretical and empirical reasons for thinking that individual differences in EI might moderate such a relationship. Consistent with the moderation hypothesis, we expected to find evidence for a significant life dissatisfaction/happiness x EI interaction for explaining suicide risk, in which the effects of reduced well-being on suicidal behaviours would be strongest in the presence of poor EI abilities.

## Method

### Participants and Procedure

Participants received a questionnaire set containing the measures assembled in this study, as well as additional ones for other scientific purposes. All participants were provided with written informed consent, which indicated that all data would be kept strictly confidential. They received no financial compensation for participation in the study. The study was carried out in accordance with the Declaration of Helsinki and ethical guidelines of the American Psychological Association. The Research Ethics Committee of the University of Málaga approved the study protocol as part of project PSI2012-38813.

Common inclusion criteria included being unemployed and actively looking for a job at the time of this survey. Therefore, no participants under 18 years of age were enrolled in this study. Approximately 70% of those approached were willing to participate. In all, 1,125 participants (506 men and 619 women) were included in the data analyses, but responses to Suicidal Behaviours Questionnaire—Revised (SBQ) for 165 participants were eliminated from the final analyses because they did not fully complete the instrument. Overall, the mean age was 35.24 years, (range 17–64 years; SD = 11.48). The educational level in the present sample was: 6% no studies, 36% primary studies, 17% incomplete secondary, 19.4% complete secondary studies, 19.2% university studies, and 2.2% post-graduate studies. The average duration of unemployment was 22.17 months (SD = 30.94 months). A total of 66% had been unemployed for more than 12 months. The marital status of the participants was: 49.9% single, 34.5% married, 8.5% separated/divorced, 1.9% widow/ widower and 5% coupled. With respect to ethnicity, all participants were White/Caucasian.

### Materials

#### Satisfaction with Life Scale [39]

This scale comprises five self-referencing statements on perceived global life satisfaction and requires subjects to rate the extent they agree or disagree with each statement on a 7-point Likert scale (1 = strongly disagree to 7 = strongly agree). Participants completed the Spanish version of the Satisfaction with Life Scale [40]. Both English and Spanish versions have shown evidence of discriminant validity and appropriate internal consistency [39,40].

#### Subjective Happiness Scale [41]

The Subjective Happiness Scale (SHS) is a four-item measurement of global subjective happiness. Each item was assessed on a 7-point Likert scale (e.g., Item 1 “In general I consider myself”: 1 = Not a very happy person to 7 = A very happy person). Higher scores reflect higher levels of subjective happiness. The SHS has shown high internal consistency, high test–retest and self-peer correlations reliability and high convergent and discriminant validity. We used a well-validated Spanish version [42].

#### Emotional intelligence

We used the Spanish Wong and Law Emotional Intelligence Scale (WLEIS) to measure EI [43]. This self-report measure is based on the definition of EI proposed by Salovey and Mayer and consists of four dimensions: self-emotion appraisal, other-emotion appraisal, use of emotion, and regulation of emotion [44]. Each subscale consists of four items with a seven-point response format ranging from 1 (strongly disagree) to 7 (strongly agree). It includes items such as, “I am sensitive to the feelings and emotions of others” and “I am quite capable of controlling my own emotions.” Respondents were asked to rate their agreement. The WLEIS elicits a global perceived EI score, which was used in this study, with higher scores indicating greater EI. The version of WLEIS has been proven to have good validity and reliability in Spanish populations [45].

#### Suicidal thoughts and behaviours

were assessed utilising the Suicidal Behaviours Questionnaire—Revised (SBQ-R) [46,47]. The SBQ-R consists of four items assessing lifetime suicidal ideation and attempt, frequency of suicidal ideation over the past year, threat of suicidal behaviour, and self-reported likelihood of future suicidal behaviour on a Likert-scale. Item scores are summed to obtain a total score. The SBQ-R has excellent reliability and validity in use with college students, as well as in clinical samples [46]. The SBQ-R was professionally translated from English to Spanish and then independently back-translated from Spanish to English.

### Data analytic plan

After calculating means, standard deviations, and internal consistency reliabilities for each scale, and computing the intercorrelations among the life satisfaction, happiness, perceived EI and suicidal behaviours, specific analyses were conducted for testing for possible moderating effects of perceived EI. Moderation analyses were conducted using the PROCESS macro [48]. Gender, age, and length of unemployment were included as control variables.

## Results

### Descriptive analyses

Pearson correlations, means, standard deviations and reliability of the different subscales used for the present sample are presented in Table 1. As expected, life satisfaction was moderately correlated with subjective happiness. Furthermore, life satisfaction and happiness were positively and moderately associated with perceived EI and negatively associated with suicidal behaviours. Finally, perceived EI correlated negatively with suicidal behaviours.

**Table 1:** Means, standard deviations, reliabilities and correlations of the variables of interest.

	1	2	3	4
1. Life satisfaction (SWLS)	--			
2. Subjective happiness (SHS)	.55**	--		
3. Emotional intelligence (WLEIS)	.38**	.39**	--	
4. Suicidal behaviours (SBQ-R)	-.28**	-.30**	-.23**	--
M	4.25	4.89	4.98	4.20
SD	1.27	1.15	.99	2.34
Alpha	.83	.74	.92	.79

Life satisfaction = Satisfaction with Life Scale (SWLS); Subjective happiness = Subjective Happiness Scale (SHS); Emotional Intelligence = Wong and Law Emotional Intelligence Scale (WLEIS), Suicidal behaviours = Suicidal behaviours Questionnaire Revised (SBQ-R).

**p < .01

### Moderator analyses

Moderation analyses were conducted to test the two hypotheses that perceived EI would moderate the relationship between (1) life satisfaction and suicide risk and (2) happiness and suicide risk, controlling for age, gender and length of unemployment. Analyses were implemented in SPSS 22.0, using the publicly available SPSS macro PROCESS [48]. This macro runs a series of OLS regressions with the centered product term representing the interaction of life satisfaction/happiness × Perceived EI as a predictor of suicidal ideation. In each case, the covariates, the predictor and moderator were mean centered, covariates were entered in step 1, the main effects were entered in step 2, and the interaction term was entered in step 3. Conditional effects at plus and minus one standard deviation around the mean of perceived EI were estimated, with confidence intervals generated at the 95% level (See Table 2).

**Table 2:** Tested moderation models with suicidal ideation as outcome predicted by perceived EI and interactions product.

	b	SE b	R^2^	Δ R^2^	95% CI
**Model 1. Life satisfaction**			.13**		
Constant	13.14	1.09			10.99 to 15.29
Gender	.11	.14			-.82 to .00
Age	-.00	.00			-.17 to .39
Length of unemployment	-.00	.00			-.00 to .00
Life Satisfaction	-1.75**	.25			-2.25 to -1.25
Perceived EI	-1.42**	.21			-1.84 to -1.00
Perceived EI x life satisfaction	.26**	.04		.03**	.17 to .36
**Model 2. Happiness**			.14**		
Constant	15.76	1.28			13.23 to 18.29
Gender	.15	.14			-.12 to .43
Age	-.01*	.00			-.02 to -.00
Length of unemployment	-.00	.00			-.00 to .00
Happiness	-2.09**	.26			-2.62 to -1.57
Perceived EI	-1.78**	.25			-2.28 to -1.27
Perceived EI x Happiness	.32**	.05		.03**	.21 to .42

Note: b = Unstandardized beta; SE b = Standard error of beta

* p<0.05;

**p<0.01

In the first analyses, no covariates effects were found. Both main effects explained a significant portion of the variance in suicidal ideation with life satisfaction and EI acting as significant predictors. When the interaction term involving life satisfaction and perceived EI was entered, it was found to account for a significant 3% of additional variance in suicidal behaviours, after partialling out the variances accounted for by both life satisfaction and perceived EI. The full prediction model (F(6, 942) = 26.02, p < .001), including covariates, life satisfaction, perceived EI scores, and the interaction term, was found to account for 13% of variance in suicidal behaviours.

In the second analysis, age showed to be a small but significant predictor. Similarly, both main effects, happiness and perceived EI, acting as significant predictors of the variance in suicidal ideation. Finally, the interaction term involving happiness and perceived EI was found to account for a significant 3% of additional variance in suicidal behaviours, after partialling out the variances accounted for by both happiness and perceived EI. The full prediction model (F(6,942) = 26.12, p < .001), including covariates, happiness, perceived EI scores, and the interaction term, was found to account for 14% of variance in suicidal behaviours.

**Fig 1:**
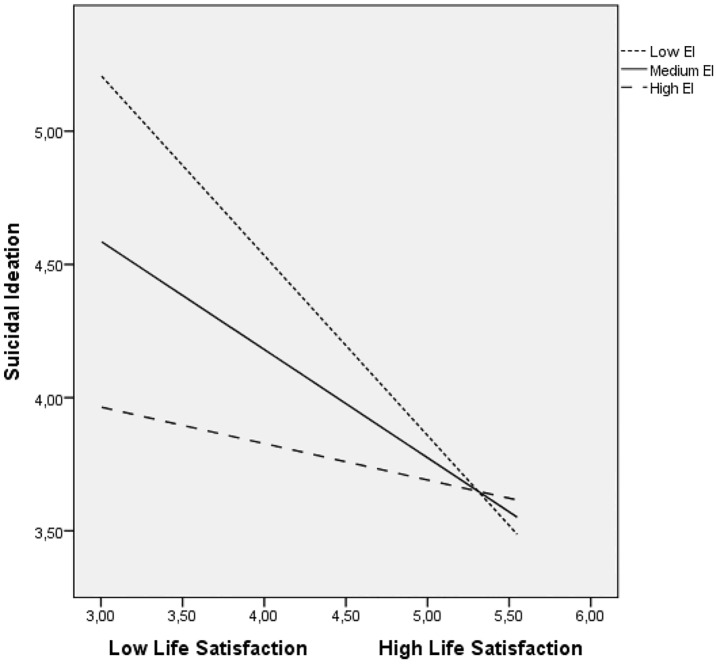
Relationship of life satisfaction and perceived EI for predicting suicidal ideation.

**Fig 2:**
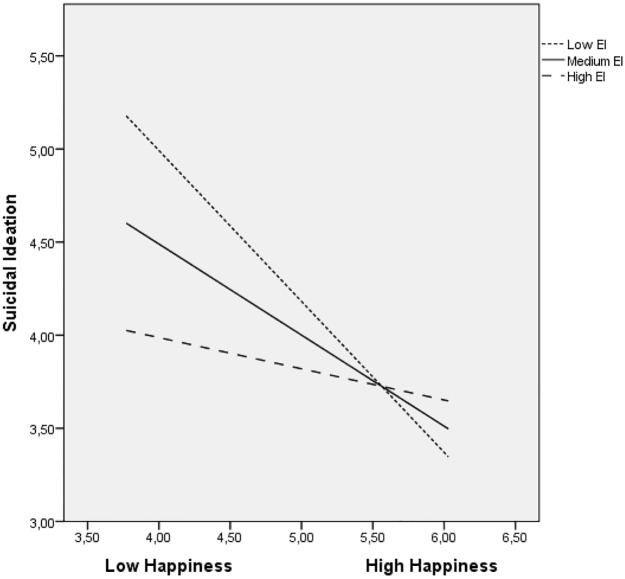
Relationship of happiness and perceived EI for predicting suicidal ideation.

Significant interactions were probed and plotted using PROCESS [48]. (see Figs 1 and 2). At low levels of both life satisfaction and happiness, unemployed individuals with low levels of perceived EI had an increased likelihood of suicidal behaviours, and those with greater perceived EI had a lower likelihood of having suicidal behaviours. In summary, individuals that were more emotionally intelligent were less likely to express suicidal ideation as a result of low levels of both life satisfaction and happiness.

## Discussion

The current study examined the relation between well-being, perceived EI, and suicidal ideation, and tested the interactive effects of EI and reduced well-being in relation to suicide risk among Spanish unemployed individuals. As mentioned earlier, although there exists evidence for the explanatory power of well-being outcomes, and particularly life dissatisfaction and low happiness, along with EI abilities in predicting suicidal ideation in college and community samples, the way in which EI might influence the relationship between reduced well-being–suicidal ideation in the unemployed populations has never been examined.

As expected, life satisfaction and happiness were found to be negatively associated with suicidal thoughts and behaviours. Besides, correlations were similar in magnitude in both dimensions. The finding on the detrimental relationship of reduced well-being on suicidal thoughts is consistent with previous empirical research [9, 19] and theory [13]. Jahoda's deprivation approach provided theoretical explanations for why unemployment may negatively impact individuals’ well-being and might cause suicide. Accordingly, the association between unemployment, unhappiness, and suicide risk might emerge from its direct effects on the individual via mechanisms such as increased probability of depressive illness, the losses of time structure, lower social status, fewer social contact or financial strain, between others [13]. Since subjective well-being has been defined as “a person´s subjective evaluation of the degree to which his or her most important goals, needs, and wishes have been fulfilled” [49], levels of life dissatisfaction due to the relative deprivation of psychosocial benefits of employment and inability to achieve some personal needs and goals are expected to influence the probability of subsequent affective and behavioral responses (suicidal ideation and behaviours). Taken together, interventions designed to reduce suicide risk may benefit from the inclusion of activities and psychological strategies to increase positive emotions and satisfaction with different domains in life [50–54].

In line with previous meta-analyses on unemployment and well-being outcomes [5,6, 35], our results suggest that the wide range of stressful problems frequently experienced by the unemployed might be related to higher levels of life dissatisfaction and reduced happiness. Additionally, in our study, EI scores were associated with higher levels of life satisfaction and happiness and lower rates of suicidal ideation. During unemployment, people with deficits in EI might experience a confluence of negative feelings associated to job loss (i.e., worry, anger, fear and hopelessness) in a way that causes them to react with suicide attempts [35]. Previous intervention studies suggest that emotional abilities can be improved, with effective benefits on psychological and physical well-being in undergraduate students [55,56] and adults [57], including positive effects on self-efficacy and employability in the unemployed population [58]. Furthermore, a recent EI intervention study among Spanish unemployed have showed that changes in emotional skills after the training were significant predictors of changes in perceived stress, mental health, somatic complaints, and mood states six months later [59]. It is plausible that similar intervention programs could be used to decrease suicidal ideation and behaviours in this at-risk population.

In terms of interaction effect, our results provide support for the moderator hypothesis. Specifically, life dissatisfaction, happiness x perceived EI interactions was found to add significant incremental validity in explaining suicidal ideation beyond what was accounted for by the main effects of each psychological construct separately. In short, we found an interactive effect of well-being indicators with perceived EI for explaining how unemployed people experience suicidal ideation. Interaction results indicated that the magnitude of the negative association between life dissatisfaction, subjective happiness and suicide risk was significantly greater in the presence of low EI than with high EI scores. Therefore, there is evidence for the buffer hypothesis that the effect of well-being outcomes on suicidal behaviours appears to be dependent on the level of EI skills. Scholars have made theoretical arguments regarding the impact of how people feel about themselves on how satisfied they report themselves as being, such that reactions to events are influenced by how emotionally worthy one views oneself to be [33,34]. The impact of low levels of life satisfaction and happiness are likely to be more profound on suicide-related conditions when individuals believe they do not have sufficient emotional resources to cope with threats [60]. Hence, it is suggested that stressful experiences such as unemployment might negatively influence individuals over time, causing them to become less satisfied with their lives and experience emotional adverse reactions including suicide thoughts and behaviours [35, 61]. However, EI abilities might modify the manner in which an unemployed views and reacts to negative mood states and life dissatisfaction associated to job loss, which may help alleviate some prevalent and dangerous behaviour problems, such as suicidal ideations and attempts [62,63]. Our findings may be particularly important for suicide prevention efforts for unemployed individuals, a specific population that is at high risk for suicidal ideation. For example, unemployed who understand appropriately negative emotions and manage the competing socio-economic demands associated to job loss appear to be protected from adverse psychological outcomes of unemployment, thereby mitigating suicide risk Our investigation also contributes to a growing body of research that investigates how levels of dissatisfaction or stress outcomes vary with regards to suicide risk due to the intervening role of personal resources, such as dispositional optimism [64], gratitude [65] or humour styles [66], between others.

It is important that the findings of the present study are considered in light of its limitations. First, the study was conducted using a convenience sample of unemployed individuals, rather than using a clinical sample. Though unemployed individuals are considered at increased risk for suicide [35], it is possible that the findings may not generalise to clinical population. Future research to determine whether the same relationships among life dissatisfaction, low happiness, EI, and suicidal ideation exist in unemployed individuals with diagnosed clinical disorders would be informative. Second, while the ordering of variables in our analyses was grounded in theory [62] and longitudinal evidence [9,10], our use of a cross-sectional design limits the interpretations of the associations and precludes any causal inference. Future studies should incorporate longitudinal research designs to examine the causal directions of these relationships during period of unemployment. Thus, building from the present findings and prior work on duration of unemployment and suicide risk [35], future researchers are encouraged to examine whether EI is more effective in buffering the reduced well-being/suicide risk relationships on certain forms of unemployment compared to others (i.e. long-duration unemployed vs. short-duration unemployed), which would increase the generalisability of the results. Third, all psychological variables were assessed using self-report measures, which might lead to common method variance problems and possible biases inherent to the use of self-report assessments. Future studies should consider employing structured/ semi-structured interviews or observers' ratings and replicate these findings using performance measures of EI.

Limitations notwithstanding, there are several implications of these findings for research and practice. Theoretically, EI might exert a positive effect on suicidal ideation by reducing the deleterious effects of negative emotions provoked by unemployment or engaging in more active coping or positive reinterpretations of stressors compared to individuals with lower EI [33]. Regarding practise, these results might be relevant because EI abilities can potentially be used as a screening assessment to identify individuals who may be at risk for experiencing suicidal ideation after becoming unemployed, improving the accuracy of diagnosing suicidal ideation and providing a target for enhancement through occupational training programmes. In short, our interaction findings suggest that for unemployed people with high life dissatisfaction and unhappiness, counselling professionals should assess their levels of emotional intelligence to determine the presence of abilities to perceive, understand and regulate effectively their emotions, as deficits in these affective process may contribute to vulnerability to suicidal ideation and attempts. Hence**,** occupational skills training might be directed at providing environmental structures and learning experiences that foster the development of a diverse repertoire of adaptive emotional skills and effective mood strategies. Such efforts might be especially targeted toward those disposed to experience life dissatisfaction and negative emotions. Counselling professionals may play an important role in developing an awareness of the relationship between emotional self-efficacy and feelings, as these relate to well-being. Therapists and other members in the area of health services and occupational counselling can assist the unemployed by teaching them to identify and cope with the emotions produced by everyday stressful events after a long period of unemployment, understanding and evaluating their feelings, and increasing the perceived belief that they can change themselves. Adaptive emotional regulation strategies should be stimulated, and negative coping patterns associated with life dissatisfaction and suicidal ideation should be prevented or challenged over the unemployment period. As underscored by social researchers, disrupting the spiralling cycle of negative and constricted emotions as a consequence of the unemployment experience can be accomplished through provision of empathic support, identification of alternatives in which the suicidal individual may believe, or re-appraisal of the negative emotions as being potentially manageable or endurable, reducing the biasing effect of negative mood on cognition [62].

In conclusion, although these findings warrant replication, our research provides some support that psychosocial and clinical interventions managing negative moods by active acquisition of specific emotional skills may be indicated as an integral part of treatment for reducing suicide risk in unemployed individuals having evident deficits in emotional skills domains [62]. Screening and assessment strategies for suicide prevention might incorporate EI ability skills as a means of identifying those unemployed individuals that are at-risk. As a challenging step in suicide research, future studies should investigate whether increasing EI abilities during group intervention serves as a promising preventative measure against suicide risk in the context of unemployment.

## Supporting Information

S1 Database (SAV)
